# Comprehensive copy number analysis of spinal muscular atrophy among the Iranian population

**DOI:** 10.1038/s41598-024-76815-x

**Published:** 2024-12-02

**Authors:** Ali Khanbazi, Maryam Beheshtian, Maryam Azad, Masoumeh Akbari Kelishomi, Fariba Afroozan, Fatemeh Fatehi, Khadijeh Noudehi, Shima Zamanian Najafabadi, Mohammadamin Omrani, Haleh Habibi, Maryam Taghdiri, Isa Abdi Rad, Shahriar Nafissi, Aria Jankhah, Hilda Yazdan, Parvaneh Daneshmand, Seyed Hosseinali Saberi, Kimia Kahrizi, Ariana Kariminejad, Hossein Najmabadi

**Affiliations:** 1 Kariminejad - Najmabadi Pathology & Genetics Center, Tehran, Iran; 2 Genetics Research Center, University of Social Welfare & Rehabilitation Sciences, Tehran, Iran; 3 Urology and Nephrology Research Center (UNRC), Shahid Beheshti University of Medical Sciences, Tehran, Iran; 4 Dr Habibi genetic counseling center, Hamedan, Iran; 5 Shiraz Genetic Counseling Center, Welfare Office, Shiraz, Iran; 6 Cellular and Molecular Research Center, Cellular and Molecular Medicine Institute, Urmia University of Medical Sciences, Urmia, Iran; 7 Neuromuscular Research Center, Tehran University of Medical Sciences, Tehran, Iran; 8 Department of Neurology, Shariati Hospital, Tehran University of Medical Sciences, Tehran, Iran; 9 Shiraz Genetic Counseling Center, Shiraz, Iran; 10 Daneshmand Medical Genetics Center, Amol, Mazandaran Iran; 11 Medical-Genetic Counseling Center, Alborz Welfare Organization, Karaj, Iran

**Keywords:** Spinal muscular atrophy, Carrier frequency, Silent carriers, *SMN*, Copy numbers, Iran, Clinical genetics, Medical genetics, Population genetics

## Abstract

Copy number variations in the *SMN1* gene on chromosome 5 are the primary cause of Spinal Muscular Atrophy (SMA) disease, characterized by muscle weakness and degeneration due to impaired alpha motor neurons in the spinal cord. To obtain a comprehensive molecular understanding of the SMA, including carriers, silent carriers, and patients in the Iranian population, we analyzed data from 5224 individuals referred to Kariminejad - Najmabadi Pathology & Genetics Center, Tehran, Iran, between 2006 and 2023 using MLPA and quantitative RT-PCR methods. The carrier frequency of SMA was estimated to be 5.55%. Furthermore, 3.06% of SMA parents (*n* = 24) had two copies of the *SMN1* gene. Among 725 patients, those with an earlier onset of SMA were more likely to have two copies of the *SMN2* gene (46.45%) and no copies of the *NAIP* gene (49.36%). Among the 654 fetal samples screened for SMA, 22.33% were found to be affected, while 3.46% of their parents tested normal. These findings are valuable for genetic counseling, carrier screening, and prenatal diagnosis of SMA in Iran. Furthermore, they underscore the importance of CNV analysis of *SMN1*, *SMN2*, and *NAIP* genes for accurate diagnosis and prognosis of SMA.

## Introduction

Spinal Muscular Atrophy (SMA), resulting from the degeneration of motor neurons within the spinal cord, is characterized by muscle weakness stemming from progressive muscle degeneration and atrophy. The estimated incidence of this condition is approximately 1 in 10,000 individuals, with a prevalence ranging from 1 to 2 per 100,000 population^1,2^. SMA is an autosomal recessive disorder caused by mutations in the survival of motor neurons 1 (*SMN1*) gene, located in an inverted, duplicated region on chromosome 5 (locus 5q13). This gene encodes a 294 amino acid protein, which, along with other proteins, constructs the SMN complex. The SMN complex is essential in assembling spliceosomal small nuclear ribonucleoproteins (snRNPs)^3,4^. The majority of SMA patients (94%), have a homozygous deletion of the *SMN1* gene, while the remaining cases exhibit inherited or *de novo* point mutations^5^. Most deletions in the *SMN1* gene involve exons 7 and 8; however, in some cases, recombination between exon 7 of *SMN2* and exon 8 of *SMN1* can lead to the formation of a hybrid *SMN* gene, where exon 7 is deleted while exon 8 remains intact^6^. Although the condition primarily results from mutations in the *SMN1* gene, other genes within the same genomic region, notably *SMN2* and the neuronal apoptosis inhibitory protein (*NAIP*), play critical roles in influencing disease severity. *SMN2* is a very similar gene to *SMN1*, with only a few nucleotide differences including two exonic variations (c.840 C > T in exon 7 and c.*239A > G in exon8). The change in exon 7 affects the splicing process, leading to truncation and instability of the *SMN2* protein^3,7^. The distinct nucleotides in these genes are targets for developing molecular genetic methods to differentiate between genes, quantify their copy numbers, and detect *SMN1* mutations. The severity of SMA is inversely related to the copy numbers of the *SMN2* gene, as it can produce a limited amount of *SMN* protein. Additionally, severe types of SMA cases often display deletions in the exon 5 of the *NAIP* gene, possibly due to unequal crossover, while milder cases usually lack *NAIP* deletions. It’s important to note that *SMN2* and *NAIP* mutations don’t cause SMA but can affect disease presentation^8–11^.

Regarding the recommendations from The American College of Medical Genetics (ACMG) advocating for population-based carrier screening for SMA^12^, several countries have contributed data regarding carrier frequency. Ethnicity emerges as a significant factor influencing allelic variations of *SMN1*, with Iran and Arabic countries demonstrating elevated carrier frequencies, while individuals of African descent exhibit a notably higher prevalence of duplicated alleles. This disparity implies a greater proportion of ‘2 + 0’ carriers within these populations, potentially resulting in a lower detection rate compared to other ethnic groups. Furthermore, previous research conducted in Iran has indicated a noteworthy elevation in carrier risk and ‘2-copy’ risk among Iranians^2^.

Given the absence of comprehensive molecular picture of SMA in Iran in the context of carrier frequency, silent carrier status, and patient diagnosis, we aimed to present the result from the molecular analysis of a large sample of individuals referred to Kariminejad - Najmabadi Pathology & Genetics Center for carrier detection, diagnosis and prenatal diagnosis which can facilitate informed decision-making and genetic counseling for families within multi-ethnic populations with a high prevalence of consanguineous marriage, such as Iran.

## Results

### Investigated individuals

**Fig. 1:**
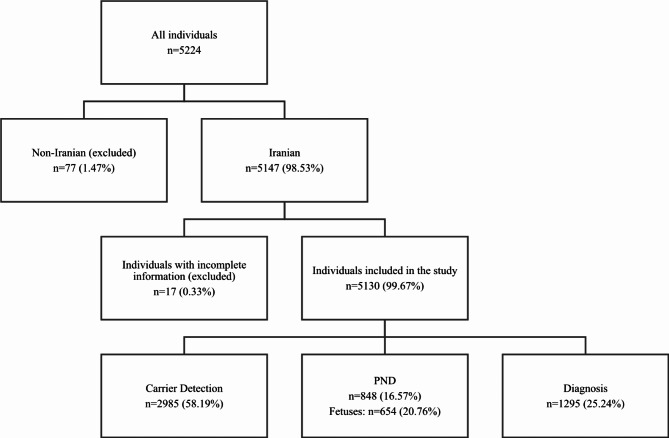
Flowchart of studied individuals.

A total of 5224 individuals were referred to our laboratory and tested for SMA between 2006 and 2023. Out of all the individuals investigated in our study, 42.92% (*n* = 2242) had a positive history of SMA in their core and/or extended family. Of note, 39.28% (*n* = 2052) were born to consanguineous parents. Figure 1 illustrates the sequential pathway followed for the filtration and categorization of individuals.

### Carrier frequency of SMA

Out of 2985 individuals referred for carrier detection, analysis of *SMN1* copy numbers among 1225 non-relative Iranian individuals (Mean age (SD) = 30 (± 7) years) with no prior history of SMA, showed that 5.55% (*n* = 68, CI = 0.95%, 4.36–7.02) had one copy of *SMN1* being a carrier for the disease. Among the remaining individuals, the majority (86.05%, *n* = 1054) demonstrated two copies of *SMN1*, while the rest of the individuals exhibited an occurrence of more than two copies. We also assessed the copy numbers of *SMN2* among the same population. Data on both *SMN1* and *SMN2* copy numbers is presented in Table 1.

**Table 1:** Distribution of *SMN1/SMN2* Copy numbers among 1225 Iranian individuals.

SMN1		SMN2	
Copy number	Frequency	Copy number	Frequency
Zero	0 (0.00%)	Zero	57 (4.65%)
One	68 (5.55%)	One	355 (28.98%)
Two	1054 (86.04%)	Two	641 (52.33%)
Three	87 (7.10%)	Three	80 (6.53%)
Four	16 (1.31%)	Four	10 (0.82%)
NA	0 (0%)	NA^*^	82 (6.69%)
Total	1225	Total	1225

* Individuals who underwent testing using Real-time PCR did not have data on *SMN2* copy numbers because only exon 7 of *SMN1* was checked.

**Fig. 2:**
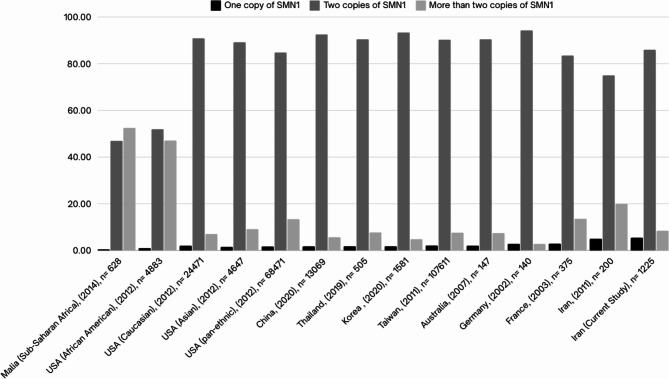
Copy number(s) of *SMN1* in normal individuals from different countries with no family history of SMA.

SMA carrier frequency across various countries is summarized in Table 2. Studies reporting the distribution of *SMN1* copy numbers among healthy individuals are presented in Fig. 2.

**Table 2:** Carrier frequency of SMA across various countries.

Country	Carrier frequency	Sample size	Technique	Reference
Iran (current study)	68 (5.55%)	1225	Real-time PCR/MLPA p021	
Iran (previous study)	10 (5.00%)	200	Real-time PCR	^13^
Saudi Arabia (2007)	9 (4.81%)	187	Multiplex-PCR	^14^
Morocco	6 (4.00%)	150	Real-time PCR	^15^
Qatar	381 (2.84%)	13,426	The *SMN* copy number caller tool using WGS data	^16^
Saudi Arabia (2022)	108 (2.57%)	4198	Multiplex PCR with Dral restriction fragment analysis	^17^
North India	16 (2.64%)	606	MLPA p060	^18^
Taiwan	2262 (2.1%)	107,611	DHPLC/ Multiplex-PCR	^19^
Korea	29 (1.83%)	1581	MLPA p460 A1	^20^
China	231 (1.77%)	13,069	Quantitative Real-time PCR	^21^
Thailand	9 (1.78%)	505	Quantitative Real-time PCR	^22^
USA (Pan-ethnic)	1162 (1.69%)	68,471	Quantitative Real-time PCR	^23^
Germany	4 (2.86%)	140	Real-time PCR	^24^
France (2003)	11 (2.93%)	375	Competitive PCR and primer extension	^25^
France (2012)	13 (2.09%)	621	Quantitative multiplex PCR	^26^
Sweden	9 (1.79%)	502	Quantitative multiplex PCR	^26^
Australia	3 (2.04%)	147	Quantitative Real-time PCR	^27^
Sub-Saharan Africa	3 (0.48%)	628	qPCR	^28^

### SMA parents with two or more *SMN1* copies

From a total of 785 parents with at least one SMA-affected child, who were considered to be obligate carriers of SMA, 96.69% (*n* = 759) had one copy, 3.06% (*n* = 24) had two copies and 0.25% (*n* = 2) had three copies of *SMN1*. Table 3 presents the proportion of SMA parents with two copy numbers of *SMN1* from various countries.

**Table 3:** Frequency of SMA parents with two copies of *SMN1* among various countries.

Country of study	Number of SMA parents with two SMN1 copies (%)	Sample size	Reference
Spain	21 (4.30%)	488	^29^
Australia (2007)	7 (5.98%)	117	^27^
China	2 (4.54%)	40	^30^
Japan	3 (4.61%)	65	^31^
Australia (2023)	9 (7.62%)	118	^32^
France	9 (4.45%)	202	^25^
Saudi Arabia	8 (5.33%)	150	^14^
North America	4 (4.00%)	100	^33^
Current study	24 (3.06%)	785	

### SMA diagnosis

A total of 1295 individuals presenting with symptoms or signs indicative of SMA were referred for diagnosis. Among them, 725 individuals were confirmed to have SMA through the identification of homozygous deletion in exon 7 of the *SMN1* gene, resulting in a diagnostic rate of 56%. Of note, 3.63% of cases (*n* = 47) exhibited heterozygous deletion of *SMN1*, while the remaining cases (40.39%) showed normal results regarding SMA.

**Fig. 3:**
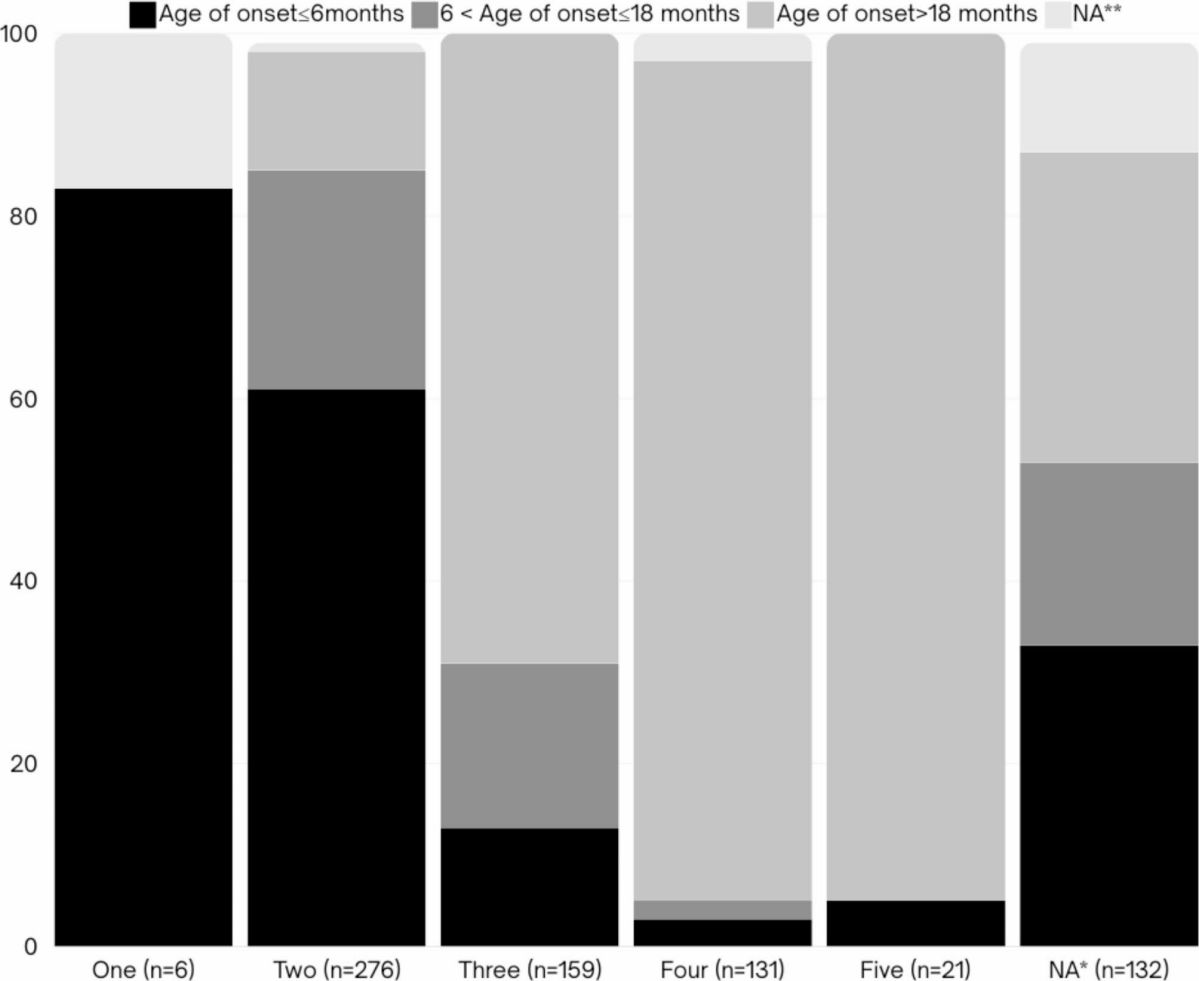
Distribution of SMA patients with various numbers of exon 7 of the *SMN2* gene. * Patients who underwent testing using Real-time PCR did not have data on *SMN2* copy numbers because only exon 7 of *SMN1* was checked. ** The age of onset for some of the patients has not been recorded.

**Fig. 4:**
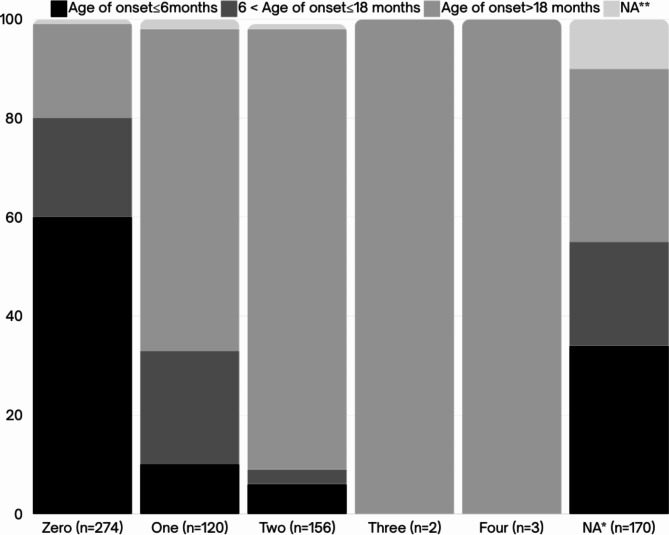
Distribution of SMA patients with various numbers of exon 5 of the *NAIP* gene. * Data on *NAIP* copy numbers for 170 SMA patients has not been documented due to either undergoing Real-time PCR testing or the absence of registered *NAIP* data. ** The age of onset for some of the patients has not been recorded.

Of all 725 patients, 81.79% (*n* = 593) were tested with MLPA and 18.21% (*n* = 132) were tested using Real-time PCR. The majority of cases (56%) were born to consanguineous marriages. Figure 3 depicts the *SMN2* gene copy numbers for affected individuals with different ages of onset, indicating that the majority of patients had two copies of *SMN2*. Figure 4, on the other hand, displays the *NAIP* gene copy numbers for patients with varying onset ages, revealing that most patients had zero copies of the *NAIP* gene.

Of note, 24 patients were found to possess a deletion in exon 7 of *SMN1* while retaining exon 8, indicating the presence of a hybrid *SMN* gene. The majority of patients with the hybrid *SMN1* gene had an age of onset above 18 months (66.66%, *n* = 16), with a smaller proportion showing symptoms under 6 months of age (20.83%, *n* = 5), and the remaining cases experiencing onset between 6 and 18 months (12.5%, *n* = 3). Information regarding the copy number of *SMN2* and *NAIP* for these patients can be found in Table 4.

**Table 4:** *SMN2* and *NAIP* copy numbers of patients with hybrid *SMN* gene (*n* = 24).

	Zero copies	One copy	Two copies	Three copies	Four copies	Five copies	Total
*SMN2* copy numbers	0 (0.00%)	1(4.17%)	2 (8.33%)	13 (54.17%)	7 (29.17%)	1 (4.17%)	24
*NAIP* copy Numbers	8 (33.33%)	8 (33.33%)	8 (33.33%)	0 (0.00%)	0 (0.00%)	0 (0.00%)	24

### Prenatal diagnosis

Data from a total of 654 fetuses were examined in our analysis. Among them, 22.33% (*n* = 146) were found to be affected, 50.15% (*n* = 328) were identified as carriers, and 27.52% (*n* = 180) tested normal.

The test results for the parents of the 146 affected fetuses from 130 families have been presented in Table 5. Notably, 3.46% of parents (*n* = 9) were tested normal for SMA.

**Table 5:** Test results for parents of affected fetuses.

Test result of parents with affected fetuses	Carrier parents	Normal parents	NA*	Total
**Frequency**	233 (89.62%)	9 (3.46%)	18 (6.92%)	260

* Data regarding parents’ test results have not been recorded in some cases.

## Discussion

In this study, we investigated individuals from across Iran, including those referred to carrier detection, parents of SMA patients, SMA patients themselves, and fetuses undergoing prenatal diagnosis. Given the diversity of our population^34^, we included carrier frequency data and information from the parents of SMA patients from various countries to provide a broader perspective on SMA across different populations. Our selection aimed to represent diverse ethnic groups, as SMA carrier frequency vary significantly among populations. We chose countries with similar cultural practices, such as high rates of consanguinity, to facilitate relevant comparisons with Iran. Additionally, we selected countries based on the availability of well-documented carrier frequency data.

After comparing our data to that of other countries to assess our status among diverse populations worldwide, we noted a higher carrier frequency rate compared to the United States, European, and East Asian populations, which confirms our earlier findings^13^. The carrier frequency in our population aligns more closely to Middle East countries which ranges from 2.57 to 4.81% (Table 2). This study highlights the significance of establishing a well-structured referral system for genetic counseling, not only in Iran but also in countries sharing a similar cultural background, particularly those with a high prevalence of consanguineous marriages. The analysis also revealed that 4.65% of normal individuals lack the *SMN2* gene (Table 1), which falls within the range of previous studies conducted elsewhere (5–15%)^3,35^.

To assess the status of silent carriers, we evaluated SMA parents to identify those with two copies of the *SMN1* gene. We found that the frequency of SMA parents possessing two *SMN1* copies is 3.06%, which may indicate the silent carrier frequency in Iran. This is also supported by our finding obtained from the investigation of the parents of the affected fetuses (3.46%) (Table 5). This result is more consistent with the reports from European, American and Asian countries (Table 3) but was lower than the previous finding by Sharifi, et al. which reported a silent carrier frequency of 11.4%^36^. A high frequency of silent carriers has been uniquely reported in African populations, which is attributed to the high copy numbers of the *SMN1* gene in this population^2,23,28^. However, a considerable dissimilarity has been noted between our population and African populations in the current study (Fig. 2) and a previous report by Mehrjoo et al.^34^, supporting the claim that our findings provide a more accurate representation of the SMA silent carriers’ status in Iran. Typically, healthy couples where one parent is a carrier are not referred to prenatal diagnosis, which poses a potential risk that should be taken into consideration by genetic counselors. Unfortunately, access to additional family members was not available in the families under investigation to discern the precise allelic phases and accurately identify silent carrier cases.

From the perspective of patient diagnosis, the second causative variant was not detected approximately in 4% of the investigated affected, indicating the need for conducting additional analysis to detect point mutations which would be beneficial in determining the precise genetic cause of the disease.

**Fig. 5:**
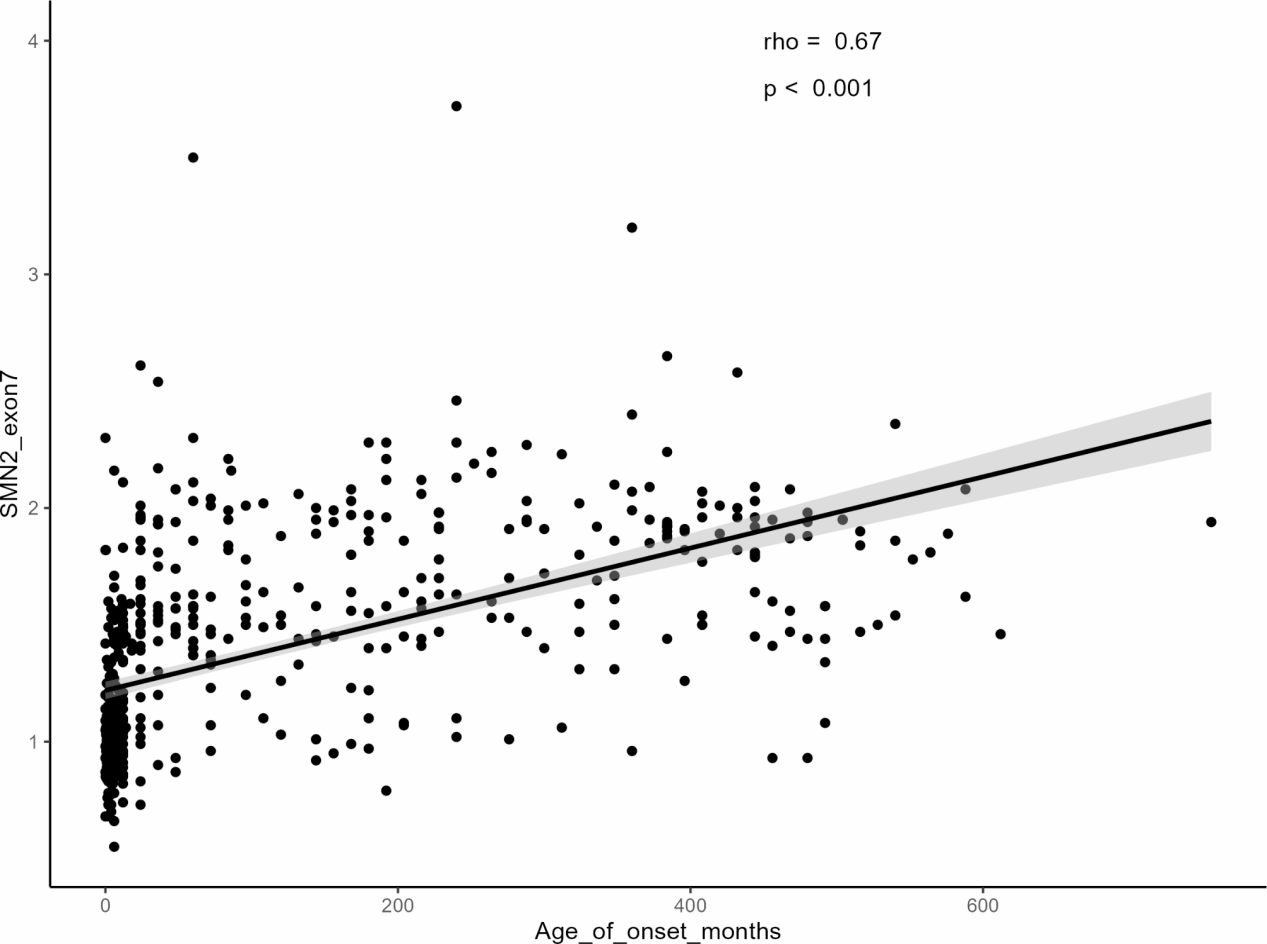
Correlation between *SMN2* copy numbers (ratio of exon7) and age of onset of the disease.

**Fig. 6:**
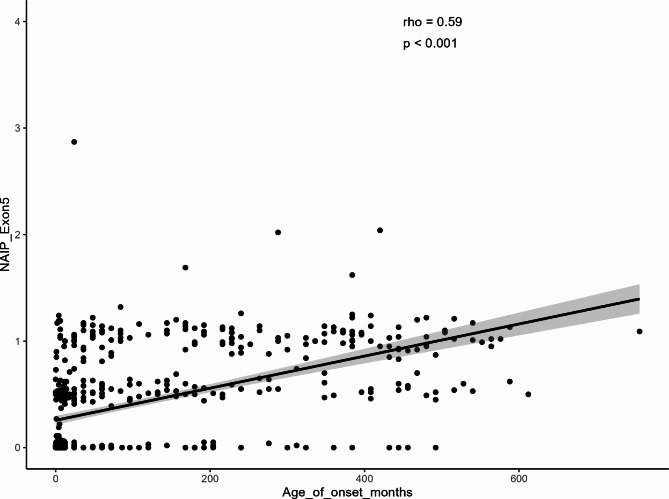
Correlation between *NAIP* copy numbers (ratio of exon 5) and age of onset of the disease.

Furthermore, *SMN2* and *NAIP* copy numbers observed in patients, demonstrated a positive correlation with the onset of the disease (Figs. 5 and 6), remaining largely consistent with previous studies^2,37,38^.

The findings regarding the hybrid *SMN* gene in our patients indicate that, according to prior reviews and previous research conducted in Iran^39^ the prevalence of individuals with the hybrid *SMN1* gene falls within the low-frequency category^40^ In contrast to earlier research^39^, our study did not show a notable correlation between the hybrid *SMN* gene and disease severity, as measured by the age of onset (Correlation coefficient: 0.071, P-Value: 0.088). This may be attributed to the fact that both studies had a limited sample size, with only 24 samples included in each study.

This report represents the largest sample size reported in Iran to date, providing valuable insights into the frequency of carriers and silent carriers, as well as the results observed in patients with SMA. These findings underscore the significance of promoting genetic counseling and carrier screening for SMA before pregnancy in Iran, along with acknowledging the potential risk of being a silent carrier. Subsequently, it’s essential to prioritize prenatal testing for couples at risk to prevent the birth of children affected by SMA. Additionally, it’s crucial to identify the copy number variations of *SMN2* and *NAIP* genes in patients, as they play a vital role in predicting prognosis and characterizing the disease phenotype.

More extensive genotype-phenotype correlations are limited due to the insufficient clinical characteristics’ data. Additionally, limited access to the investigated families prevented us from confirming the status of silent carriers.

## Materials and methods

### Subject

We analyzed data of 5224 individuals referred to Kariminejad - Najmabadi Pathology & Genetics Center for SMA carrier detection, and patient diagnosis between 2006 and 2023 by physicians. Data from non-Iranian individuals (*n* = 77, 1.47%) and individuals with inadequate information (*n* = 17, 0.33%) were excluded from the study dataset. The flowchart depicting the studied population has been shown in Fig. 1.

### Methods of testing

**Fig. 7:**
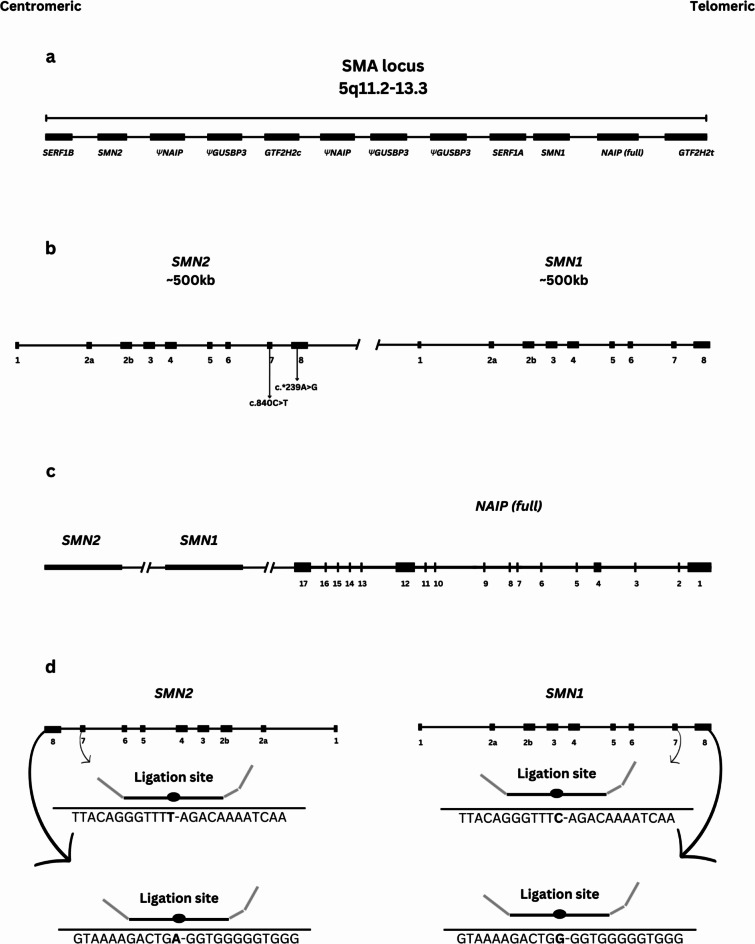
Schematic diagram of the SMA locus.** a**: expansion of the SMA locus on the long arm of chromosome 5, including *SMN1*, *SMN2*, and *NAIP* genes, as well as nearby genes and pseudogenes in this region^42^.** b**: *SMN1* and *SMN2* genes and two nucleotide differences in exons 7 and 8 of *SMN1* and SMN2*.** c**: the *NAIP* gene and its position near the *SMN1* and *SMN2*. **d**: Mechanism for detecting *SMN1* and *SMN2* copy numbers using the MLPA technique. The ligation site of each probe is located in the different variants of the two genes on exons 7 and 8. * The exon numbering in this schematic diagram follows the traditional format, rather than the sequential 1 to 9 numbering used in online databases for the exons of the *SMN1* and *SMN2* genes.

The majority of the samples (*n* = 4095, 78.38%) were tested using the Multiplex Ligation-dependent Probe Amplification (MLPA) technique, following the manufacturer’s instructions as outlined in the MRC Holland website (https://www.mrcholland.com/product/P021/634). The MLPA analysis utilizes a set of 32 specific probes targeting different regions of the SMA locus. Specifically, two probes target exon 7 and exon 8 of the *SMN1* and *SMN2* genes. Detection accuracy for copy number variations and gene conversions is remarkably reliable, with both analytical sensitivity and specificity exceeding 99%. The ligation site of these probes is located at different nucleotides between the two genes. This difference in the ligation site allows the MLPA assay to distinguish *SMN1* from *SMN2*, as the probes will only ligate and amplify their respective target sequences (Fig. 7). The PCR products obtained from the MLPA analysis were subsequently analyzed using Coffalyser.NET software, which is also developed by MRC-Holland. The final ratios were determined by comparing each sample to reference samples to compute the copy numbers. Internal validation with 16 DNA samples from healthy individuals was conducted to ensure a standard deviation of ≤ 0.10 for all reference probes.

Before to the widespread adoption of MLPA for copy number variation analysis, the earlier samples (n = 1129, 22.61%) were tested using Real-time PCR with the delta-delta Ct method to determine the copy numbers of exon 7 of the *SMN1* gene with the specificity of 100% and a sensitivity of 96.2%^41^. with SYBR green I dye. The real-time PCR assay utilized primers specifically designed to amplify the *SMN1* gene. To differentiate *SMN1* from the highly similar *SMN2* gene, the 3’ ends of the primers are designed to target *SMN1*-specific sequences - in exon 7 (Forward primer: 5’-CCTTTTATTTTCCTTACAGGGTTTC-3’, reverse primer: 5’-GATTGTTTTACATTAACCTTTCAACTTTT-3’). The specificity of the *SMN1* primers was confirmed with the *Albumin* gene in samples from both patients and normal individuals (Forward primer: 5’-AGCTATCCGTGGTCCTGAAC-3’, reverse primer: 5’-TTCTCAGAAAGTGTGCATATATCTG-3‘). To validate our test results, 20 SMA patients, 20 obligate carrier couples, and 20 healthy individuals from the normal population were tested. The methods employed in this study followed the relevant guidelines and regulations.

### Statistical analyses

Data filtration and statistical analyses were performed in RStudio software, version 2023.09.1 + 494. Statistical significance was determined at a threshold of *p* < 0.05. To estimate the carrier frequency among the Iranian population, a confidence interval (CI) of 95% was employed. Non-parametric Spearman’s correlation coefficient (rho) was employed to examine the relationship between copy numbers of *SMN2* and *NAIP* genes and the age of onset of the disease as well as assess the correlation between the presence of a hybrid *SMN* gene and the age of onset of the disease.

## Data Availability

The datasets generated during and/or analyzed during the current study are available from the corresponding author on reasonable request.
